# Symptom Presentation among Women with Suspected Ischemia and No Obstructive Coronary Artery Disease (INOCA)

**DOI:** 10.3390/jcm12185836

**Published:** 2023-09-08

**Authors:** Yasmeen K. Taha, Jennifer R. Dungan, Michael T. Weaver, Ke Xu, Eileen M. Handberg, Carl J. Pepine, C. Noel Bairey Merz

**Affiliations:** 1 College of Medicine, University of Florida, 1600 Southwest Archer Road, Gainesville, FL 32610, USA; yasmeen.taha@medicine.ufl.edu (Y.K.T.); eileen.handberg@medicine.ufl.edu (E.M.H.); carl.pepine@medicine.ufl.edu (C.J.P.); 2 College of Nursing, University of Florida, 1225 Center Drive, Gainesville, FL 32610, USA; michael.weaver@ufl.edu; 3 Department of Health Outcomes and Biomedical Informatics, University of Florida, Gainesville, FL 32611, USA; k.xu@vumc.org; 4 Barbra Streisand Women’s Heart Center, Cedars-Sinai Medical Center, Los Angeles, CA 90048, USA; noel.baireymerz@cshs.org

**Keywords:** symptoms, women, female, coronary ischemia, INOCA, no obstructive CAD

## Abstract

Identifying ischemic heart disease (IHD) in women based on symptoms is challenging. Women are more likely to endorse non-cardiac symptoms. More than 50% of women with suspected ischemia have no obstructive coronary disease (and thus, INOCA) and impaired outcomes during follow-up. We aimed to identify symptoms having predictive capacity for INOCA in women with clinical evidence of coronary ischemia. We included 916 women from the original WISE cohort (NCT 00000554) who had coronary angiography performed for suspected ischemia and completed a 65-item WISE symptom questionnaire. Sixty-two percent (*n* = 567) had suspected INOCA. Logistic regression models using a best subsets approach were examined to identify the best predictive model for INOCA based on Score χ^2^ and AICc. A 10-variable, best-fit model accurately predicted INOCA (AUC 0.72, 95% CI 0.68, 0.75). The model indicated that age ≤ 55 years, left side chest pain, chest discomfort, neck pain, and palpitations had independent, positive relationship (OR > 1) to INOCA (*p* < 0.001 to 0.008). An inverse relationship (OR < 1) was observed for impending doom, and pain in the jaw, left or bilateral arm, and right hand, interpreted as INOCA associated with the absence of these symptoms (*p* ≤ 0.001 to 0.023). Our best-fit model accurately predicted INOCA based on age and symptom presentation ~72% of the time. While the heterogeneity of symptom presentation limits the utility of this unvalidated 10-variable model, it has promise for consideration of symptom inclusion in future INOCA prediction risk modeling for women with evidence of symptomatic ischemia.

## 1. Introduction

Coronary artery disease (CAD) is the leading cause of death in women, and it is estimated to affect about 67 million women globally [1]. However, in-hospital mortality is higher in women than in men [2]. For decades, CAD was defined according to the degree of obstruction limiting epicardial coronary blood flow. Data suggest that around 3 to 4 million Americans with symptoms and/or signs suggestive of myocardial ischemia who undergo angiography have no obstructive CAD (INOCA), with a higher prevalence in women versus men [3]. Among men and women who undergo non-invasive testing for evaluation of ischemia, approximately 9% are deemed high risk (using test-specific parameters) and about 30% of those in the high-risk category have evidence of CAD with no significant obstruction [4].

As described in the 2021 AHA/ACC Guideline for Evaluation and Diagnosis of Chest Pain [5] and by others [6,7,8], chest symptoms (commonly known as “typical chest pain”) are the most prevalent presenting symptoms among both men and women (namely, chest pain located in central chest, and pain nature as ache, pressure, tightness, or discomfort). Evidence is growing that supports the suggestion that women with suspected INOCA tend to experience worse quality of life, greater physical limitation, and 5- and 10-year major adverse cardiac event (MACE) risks, which include death, heart failure, hospitalization, non-fatal myocardial infarction, non-fatal stroke, and late revascularization [9,10]. We have previously reported in the WISE cohort that women presenting with symptoms and/or signs of ischemia (suspected INOCA) had associated increased hazards of 10-year cardiovascular death and myocardial infarction (*p* < 0.01) [10].

The presence of non-cardiac symptoms in patients with suspected INOCA contributes to underdiagnosis, referral delays, and worse outcomes in women when compared with men [11]. Non-cardiac symptoms commonly described by women are epigastric pain, nausea, vomiting, extreme or unusual fatigue, and subscapular discomfort [5,12,13]; these are also extensively described in the well-validated McSweeney Acute and Prodromal Myocardial Infarction Symptom Survey MAPMISS tool [14,15,16]. To our knowledge, the difference in symptom presentation among women with obstructive versus no obstructive CAD in a diverse ethnic sample has not yet been reported, nor has a symptom-based prediction model for INOCA been tested. Accordingly, the aim of this analysis was to identify a set of symptoms predictive of no obstructive CAD in women presenting with symptoms and signs of ischemia with suspected INOCA.

## 2. Methods

### 2.1. Study Population

The Women’s Ischemia Syndrome Evaluation (WISE, NCT00000554) [17] original cohort recruited 944 women from 7603 screened who were referred for coronary angiography based on symptoms and signs of ischemia between 1996–2001. Consenting women received established standard-of-care evaluation for symptoms and signs of suspect coronary ischemia prior to and during their participation in the study. Clinical signs refer to clinical observations which may include family history, laboratory test results, electrocardiogram results, and other forms of invasive or non-invasive clinical testing. Patient care was not impacted by the study protocol. Physician cardiologists conducted their usual standard of care and angiogram referral process, as informed by multiple patient and clinical factors. For the current analysis, 28 were eliminated due to “uncertain” or “missing” data for the INOCA variable, leaving 916 women.

### 2.2. Baseline Characteristics

Baseline physical examination, demographic data, and a WISE medical history evaluation questionnaire (70-questions) on patient history and cardiovascular risk factors such as diabetes mellitus, hypertension, hyperlipidemia, peripheral vascular diseases, renal disease, and others were obtained at initial evaluation, as published [17].

### 2.3. Assessment of Symptoms

Symptoms were self-reported by all patients prior to or immediately following coronary angiography. All patients completed a detailed, standardized chest pain assessment questionnaire that evaluated: (1) symptoms experienced that led to having this evaluation, which included pain, headache, fatigue, cough, dizziness; (2) location of pain: chest, arm, epigastric, back, neck, hand or jaw pain with specification of the side (Right, Left or Central); (3) description of the sensation: aching, pressure, knife like, burning, etc.; (4) intensity of the sensation on a scale from 1 to 5, with 1 described as tolerable and no relief needed and 5 described as non-tolerable and not relieved with usual measures; and (5) what causes or relieves the sensation. The patient could mark one or more of the above questions. Of note, the 59-item symptom questionnaire was only collected for research purposes; responses to the questionnaire were not used in standard of care evaluation or clinical decision-making for patients who participated in the study. Ninety-eight percent of participants had complete symptom data across 59 variables, with only 20 out of 916 (~2%) having missing data.

### 2.4. Coronary Angiography

All participants underwent coronary angiography at initial enrollment and angiograms were masked to other patient data and evaluated for CAD in the Angiographic Core Lab. No obstructive CAD was defined as “normal” coronary arteries or <50% luminal stenosis in any epicardial artery [5]. Alternatively, obstructive CAD was defined as ≥50% stenosis, per the WISE original cohort definition [17].

### 2.5. Statistical Analysis

A total of 57 symptom characteristics plus age ≤ 55 and body mass index were available for selection. Satterthwaite independent *t*-Tests were used to compare means between obstructive and INOCA groups. Contingency tables using Fisher’s Exact test (2 × 2 tables) or likelihood ratio chi squared (tables greater than 2 × 2) with Monte Carlo calculation of exact *p* values were used to compare distributions of nominal variables between obstructive and INOCA groups. The SELECTION = SCORE option was used in SAS 9.4 (SAS Institute, Cary, NC, USA) PROC LOGISTIC [18] to identify the best multivariable predictive logistic model. That technique produces a list of the best (that is, having the largest Score χ^2^ value) models containing 1, 2, 3, and so on, predictor variables up to the model containing all available predictors (59 in this data set). Corrected Akaike Information Criterion (AICc), and Schwarz Criterion (smaller values are better for both criteria) were used to compare performance of the resulting 59 models and identify the best performing model for predicting no obstructive CAD [19,20]. All analyses were carried out using SAS version 9.4. Goodness of fit for logistic models was evaluated using the Hosmer and Lemeshow Goodness of Fit Test [21], and observation influence and tenability of statistical model assumptions were evaluated using diagnostic plots produced by PROC LOGISTIC. Profile likelihood 95% confidence intervals (CI) [22] were calculated for odds ratios (OR) based on the identified best predictive model.

## 3. Results

### 3.1. Baseline Characteristics

Among the 916 women, 567 (62%) had no obstructive CAD consistent with suspected INOCA. Pertinent descriptive statistics for the sample, by INOCA status, appear in Table 1. Compared with women with obstructive CAD, women with INOCA were younger and had fewer cardiovascular (CV) risk factors, such as hypertension, diabetes mellitus (DM), and dyslipidemia. However, they were more likely to have polycystic ovary disease (PCO), history of depression, mitral valve prolapse (MVP), and migraine headache. Conversely, women with obstructive CAD were more likely to have a history of prior myocardial infarction (MI), congestive heart failure (HF), and presence of menopause than women with INOCA. Reports of unstable angina in the last 6 weeks and recent acceleration of anginal pain were significantly higher in women with obstructive CAD.

### 3.2. Symptom Descriptive Comparisons

#### 3.2.1. Presenting Symptoms

Compared with the obstructive CAD group, women with INOCA had a significantly higher frequency of the following symptoms (Table 2): chest pain, chest pressure, shortness of breath, general chest discomfort, chest tightness, fatigue, weakness, or faintness, palpitations or rapid heart rate, neck pain, and abdominal pain.

#### 3.2.2. Pain Location, Described Sensation and Aggravating & Relieving Factors

Women with INOCA significantly endorsed left chest and left or central neck pain more frequently than women with obstructive CAD (Table 3). A majority of both groups noted the following descriptions for their anginal symptoms: pressure, discomfort, pain, or tightness; however, significant between-group differences were not observed. Seventy-four percent of women with obstructive CAD reported that their doctor said the feeling (sensation) was heart related, compared with only 55% of women with INOCA (*p* < 0.001). Most women among the INOCA and obstructive groups described their symptoms as aggravated by lower body exertion or emotions, but there were no significant between-group differences for aggravating factors. Both groups similarly experienced symptom relief with rest. Nitroglycerine alleviated symptoms more often in those with obstructive CAD compared with women with INOCA.

#### 3.2.3. Duration, Intensity, and Symptom Response

There was a statistical trend for between-group differences in the duration of symptoms (Table 4). Regardless of disease type, 95–98% of women responded to their symptoms by seeking medical care due to discomfort. More women with INOCA (42%) responded by disengaging themselves from their symptoms by doing or thinking about something else, compared with only 33% of women with obstructive CAD.

### 3.3. Best Predictive Model for Presence of INOCA

**Figure 1:**
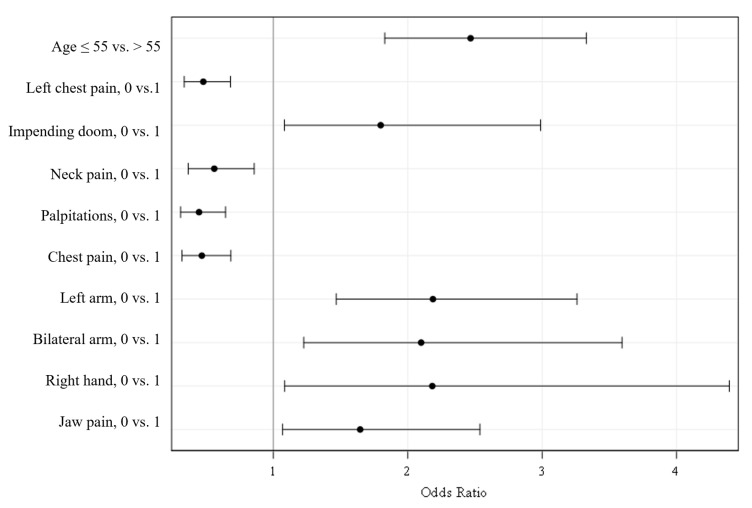
Odds Ratios with 95% Wald Confidence Limits. Forest plot of predictive factors associated with nonobstructive coronary artery disease in women with suspected ischemia. Odds ratio (OR), 95% Profile Likelihood Confidence interval.

**Figure 2:**
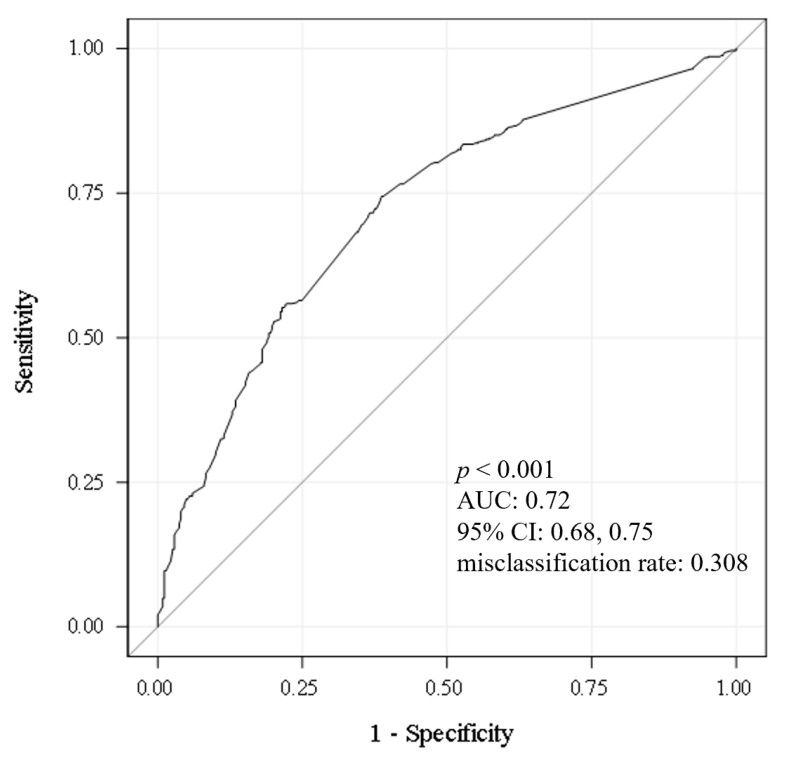
Receiver-Operating Curve for 10-Predictor Model. AUC: area under the curve, CI: confidence interval.

Logistic regression models containing from 1 to 59 (number of predictor variables available) were ordered based on score χ^2^ and evaluated using AICc (corrected Akaike Information Criterion) and Schwartz’s Criterion. Models containing between 8 and 14 predictors, with score χ^2^ values ranging from 108.3 to 122.9, were selected for preliminary evaluation. Models with between 11 and 13 predictors were eliminated due to lack of model fit to the data (Hosmer–Lemeshow *p* value < 0.05). Of the remaining models, models with 10 and 14 predictors had the lowest AICc (1109 and 1108, respectively). The model with 10 predictors had the smallest Schwartz criterion value (1162 versus 1180) and exhibited a similar area under the receiver operating (ROC) characteristic curve (AUC) of 0.720 compared with 0.726 for the 14-predictor model. Based on these results, the model containing 10 predictors was selected as the final model. Results based on that 10-predictor model are presented in Table 5 and Figure 1 and Figure 2. Of note, the 10-predictor model was generated after study closure, therefore, the model did not impact patient standard of care evaluation or clinical decision-making.

Table 5 summarizes the selected model for discriminating INOCA (overall model χ^2^ = 130.9, DF = 10, *p* < 0.001; AUC: 0.72, 95% CI: 0.68, 0.75; misclassification rate: 0.308). Each variable in that 10-predictor model demonstrated an independent linear association with INOCA, controlling for all other variables in the model. Age ≤ 55 years and presence of self-reported symptoms of palpitations, chest discomfort, pain in left side of chest, and neck pain were positively related to INOCA (odds ration [OR] > 1). Conversely, absence of discomfort in the left arm, bilateral arms, right hand, impending doom, and jaw pain were associated with INOCA (OR < 1). Figure 1 represents the effect sizes and directions for each independent predictor. Figure 2 shows the ROC curve for the selected model containing these variables.

### 3.4. Post hoc Analysis: Timing of Symptom Data Collection

The accuracy of patient recall of symptom experiences can affect the internal validity of symptom data for prediction modeling, such that time delays in data collection reduce the accuracy of self-report. The primary study’s protocol allowed for symptom data collection either before (*n* = 98) or just after coronary angiography (*n* = 559). A total of 259 participants were unable to be included in this post-hoc analysis due to missing values for the time of data collection variable. To evaluate the potential influence of recall bias, we identified a best-fit model of INOCA with the subset of 559 participants who had symptom data collected after coronary angiography. An 8-predictor model emerged as the best-fit (overall model χ^2^ = 63.6, DF = 8, *p* < 0.001; AICc 672.6, AUC 0.697). Only two of the predictors (right hand and jaw pain) from the 10-variable model were not retained in the 8-variable model for the group whose data were collected post-angiography.

## 4. Discussion

We identified a best-fit predictive model of 10 variables (age and nine symptoms) having 72% accuracy for the presence of INOCA. AUC values between 0.70 and 0.80 are considered an ‘acceptable’ level of discrimination, having room for improvement. Each of the 10 variables is an independent predictor of INOCA, controlling for all others in the model. Age ≤ 55 years, and the presence of self-reported symptoms of left side chest pain, chest discomfort, neck pain, and palpitations, demonstrated independent, positive relation to no obstructive CAD status (OR > 1). Conversely, we identified an inverse relation to no obstructive CAD for symptoms of impending doom, and for pain in the jaw, left or bilateral arm, and right hand (OR < 1), meaning that the absence of these symptoms was associated with the presence of INOCA.

In this diverse sample of nearly 1000 women from the WISE original cohort, we also observed important demographic, risk factor, and symptom differences between women with and without obstructive CAD. Women with no obstructive CAD consistent with INOCA were more likely to be younger and have fewer CV factors (better HDL cholesterol and lower frequencies of hypertension, DM, and dyslipidemia). Women with obstructive CAD were older (thus more women reported menopause), had significantly more CV risk factors, MI, HF, unstable angina in the last 6 weeks, and recent acceleration of angina compared with their no obstruction counterparts. More women with INOCA in our cohort reported PCO, depression, mitral valve prolapse, and migraine headaches.

We found no direct comparison of our 10-symptom, best-fit model predicting the presence of INOCA, nor could we find any reports of symptom cluster modeling for this purpose. A majority of symptom studies in CHD focus on symptom differences or clusters between men and women, or focus on predicting or discriminating endophenotypes of obstructive CAD, events such as MI, or quality of life [23]. We chose to model INOCA as the outcome because, clinically, INOCA presentation is less recognizable by the public and by providers and is more difficult to diagnose, yet is associated with MACE and poor outcomes, particularly among women. Thus, we set out to address a major gap in knowledge about the ability of symptoms to predict INOCA status, and to secondarily characterize whether and how symptoms may differ between INOCA and obstructive disease among women, who are the most at-risk group for INOCA and for cardiac health disparities. The closest comparative study in the literature comes from an evaluation of 1559 male and female participants of the Korean Women’s Chest Pain Registry (KoROSE) [24]. In this descriptive study, 785 Korean women designated as “non-obstructive CAD” were compared to 250 Korean women with obstructive CAD. Of importance, the KoROSE study reports only descriptive comparisons and does not attempt the predictive model-building unique to our work. In our 10-variable model, the presence of left side chest pain, chest discomfort, neck pain, and palpitations demonstrated significant prediction of INOCA, with significantly increased frequency among our females with INOCA versus obstructive CAD. In comparison, the KoROSE study only observed a significantly higher frequency for palpitations (*p* = 0.003) and a trend for left sided chest pain (*p* =0.055) among females with INOCA [24]. Unlike our results, they reported no between-group difference for neck pain (*p* = 0.342), and they did not test an equivalent of “chest discomfort”. Our model also included five symptoms for which their absence (OR < 1) was predictive of INOCA (impending doom, jaw pain, left or bilateral arm pain, and right hand pain). The KoROSE study only reported a non-significant between-group difference for left arm pain; the other four symptoms were not studied. The most likely reason for these discrepancies is that the KoROSE study defined non-obstructive CAD as any level of coronary stenosis < 70% in proximal, middle, or major branch coronary vessels among people with angina [24], whereas we used a 50% stenosis cutoff. Their inclusion of 50–70% stenosis for the non-obstructive phenotype increased the heterogeneity in the variable and possibly reduced the likelihood of identifying significant differences.

Our secondary findings include between-group differences for additional symptoms that did not appear in the best-fit predictor model, some of which are also comparable to the KoROSE study. Unlike our significant findings for increased frequency of chest pressure, shortness of breath, and fatigue/weakness/faintness among women with INOCA (all having *p* < 0.001), as well as a higher frequency trend for symptom aggravation by very hot/cold weather among women with obstructive CAD (*p* = 0.025); women of the KoROSE study had no differences between groups for pressure (*p* = 0.513), dyspnea (*p* = 0.259), syncope (*p* = 0.552), and aggravation by low temperature (*p* = 0.225) [24]. Dyspnea was significantly associated with INOCA among both men and women (*p* < 0.001) in the CIAO-ISCHEMIA study [25], and UK investigators [26] reported a statistical trend for shortness of breath among females with INOCA (12%); however, their comparison group was men with INOCA (6.6%; *p* = 0.046), using a <70% cutoff [26]. Our finding of significantly increased fatigue/weakness/faintness among women with INOCA is echoed similarly in a secondary analysis of vital exhaustion among 3656 women with INOCA from the Danish prospective multicenter iPOWER study [27]. Vital exhaustion is defined as “a state which is present when an individual not only complains of unusual fatigue and decreasing energy but also by feeling dejected or defeated…feeling exhausted when waking up is highly characteristic of this condition” [28]. Bechsgaard and colleagues [27] reported that the risk of severe vital exhaustion was more than 3-fold higher among women with chest pain and INOCA compared with asymptomatic women (OR 3.3, 95% CI 2.5 to 4.4), independent of age and risk factors, and was distinct from depression in symptomatic women. Importantly, the 37 items on the vital exhaustion instrument were selected in psychometric analysis for their joint prediction of MI [28].

Our secondary, between-group observations that common chest symptoms were the most frequently reported (>40% frequency, regardless of obstruction status) are incidental to the WISE primary study inclusion criteria (presence of angina or suspect ischemia). Therefore, we expected to observe high frequencies of chest-related symptoms among both groups, as consistent with a recent 27-study meta-analysis concluding that chest pain was the most common reported symptom by both sexes [29]. Our moderate to high rates of non-chest symptoms, such as fatigue, nausea, neck and back pain, and palpitations, across all females analyzed were also consistent with the literature [29,30]. Regarding our between-group demographic and risk factor differences (Table 1), our INOCA subset of women tended to be younger and also to report significantly more symptoms compared with those with obstructive CAD (represented by higher symptom frequencies across a majority of the symptoms). This is consistent with a systematic review of symptom clusters in CV disease, which demonstrated that younger people tend to report more symptoms than older people [23]. Therefore, the symptom frequencies observed in our younger INOCA subset may be a product of age differences. Women in our sample with INOCA reported significantly higher frequencies of PCO, depression, mitral valve prolapse, and migraine headaches compared with women with obstructive disease. The greater prevalence of these concomitant conditions among women in our sample who also had a reduced prevalence of traditional CHD risk factors of hypertension, DM type 2, and dyslipidemia (Table 1), presents a potential clinical concern, in that the symptoms of PCOS, depression, valvular disease, and migraine could cloud the presentation of ischemia and may increase the likelihood of swaying the differential diagnosis away from INOCA. This hypothesis remains untested. These conditions have been associated with CV disease but are rarely studied in the context of INOCA. PCO is associated with increased risk for CAD [31,32]. Depression, as a psychological stressor, has been implicated as both a cause and consequence of microvascular dysfunction [33]. Valve disease associated with thromboembolism (such as mitral valve disease) has also been associated with MI and no obstructive disease (MINOCA), most notably among women [34]. Migraine headaches among women have been associated with MACE among WISE women with signs and symptoms of ischemia [35] and among male-female cohorts [36,37].

Clinicians in urgent care settings rely on presenting symptoms as one of the major criteria, along with other clinical signs and testing, to diagnose ischemic coronary syndrome. Patients also recognize chest pain as an indication to seek medical care; presenting with non-cardiac symptoms of coronary ischemia without obstruction can complicate triage and diagnosis. In the Variation in Recovery: Role of Gender on Outcomes of Young AMI Patients (VIRGO) study, women who had non-chest pain symptoms were more likely to be told that their symptoms were not cardiac related [30]. Similarly, we found that, even when 95% of women with INOCA did report chest-related symptoms, they were less likely to be told that their symptoms were heart-related by a doctor (*p* < 0.001). This is of concern, as another study showed that, despite having symptoms, most women were not diagnosed with CAD before having an acute MI [38]. In the female participants of WISE specifically, women having symptoms and/or signs of ischemia with INOCA have worse outcomes (nonfatal MI or cardiovascular death) [10]. Moreover, commonly used CV disease risk scores failed to accurately predict the observed cardiovascular disease rates in women with INOCA in a more recent comparative analysis of WISE participants [39], emphasizing the need for testing risk models which incorporate female- and/or INOCA-specific factors which may include symptom predictors.

### Strengths and Limitations

The main strengths of this study include the presence of phenotyping for no obstructive CAD (INOCA), obstructive CAD, and uniquely extensive cardiac and non-cardiac symptom data. Another strength is the completeness of symptom data for nearly 1000 participants on 59 symptom predictors. Importantly, none of the 916 participants had missing values and therefore contributed 100% complete data for the 10 variables in the selected best-fit model.

We also acknowledge limitations. We note the relatively low frequency (<20%) of certain predictor variables in our best-fit model and the potential for concern about less-frequent and/or negative predictors on discrimination of the INOCA outcome. However, a variable’s frequency alone does not determine its importance; its association with the outcome—whether the effect direction is negative or positive—and its ability to improve model performance matter. Using less frequent variables in a prediction model does not necessarily make it less strong, especially if the model’s Area Under the Curve (AUC) achieves the 0.75 threshold, indicating how well the unique combination of variables in the model distinguishes between different groups (e.g., INOCA and Obstructive status). Even less frequent variables can have a significant impact on the model’s predictive power if those variables provide unique and relevant information. In predictive modeling, a balanced selection of variables that collectively capture different aspects of the problem is important. Including less frequent variables can add diversity to the model and contribute to a more comprehensive understanding of the relationships. However, there are some limitations to predictive model building regarding potential for overfitting, external validity, and interpretability. Including an excess of variables, especially if they are rare or highly specific, can increase the risk of overfitting, where the model fits the test data well but may not do so in a new dataset. Including less frequent variables may make the model more complex and less interpretable. Thus, future external validation is necessary to evaluate overfitting and generalizability, as well as to optimize interpretability. INOCA phenotype heterogeneity in research, clinical guidelines, and symptom studies limits the comparability of our results. The definition of obstructive CAD varies across studies, with thresholds of 50% and 70% used for obstructive CAD [40,41,42]; however, in our study, because the flow limitation can be associated with various degrees of stenoses, we have retained the initial WISE definition ≤50% as they are not flow limiting [17]. It is now recognized that the minority of female patients have obstructive CAD at diagnostic invasive coronary angiography or coronary computed tomography angiography (CCTA). However, that conclusion arose from the WISE original cohort of almost 1000 women. Our current INOCA findings are relevant to today’s patients with signs and symptoms of ischemia without obstructive coronary disease phenotype. While the sole comparative study (KoROSE [24]) evaluated a larger sample of females with no obstructive CAD (785 compared to our 567 women), their study represented Korean women, whereas our sample was representative of women from North America, including approximately 20% Black/African American women. Others have reported significant symptom clusters predicting outcomes among Black/African American women of the WISE cohort [36,37,43,44,45,46,47,48], although their research did not evaluate INOCA status. The inter-individual variability of symptom experiences and the heterogeneity of cardiac and non-cardiac symptoms limit the clinical utility of using age and symptoms for diagnosis of INOCA. Our pilot observations reveal that inclusion of symptoms may contribute discriminatory value in future prediction model-building for INOCA. The implications of this work reflect pilot observation in informing future prospective study design with hypothesis testing. The scientific community may desire an evaluation of our model’s predictive capacity compared to INOCA testing or imaging standards; however, no such standards exist. WISE investigators have assessed six popular risk scores frequently used for CAD assessment [39] and applied them to the women in this report. All of these scores significantly underestimate the adverse outcomes risk among these women. Additionally, WISE investigators explored use of nuclear, echo and cardiac MRI ischemia testing in the original cohort; however, this did not yield consistent findings. With exercise treadmill testing (ETT), even using a modified exercise protocol, only a minority could achieve much stress [44] and these results were non-diagnostic. They also explored use of cardiac PET and found that when perfusion defects were detected the defects were often very heterogenous and difficult to assess [45]. They also explored use of dobutamine stress echocardiography [46] with similar non-diagnostic results. Most recently, in an Ancillary Ischemia study, investigators [47] used exercise testing with echo imaging and concluded that, “There was no relationship between extent of nonobstructive atherosclerosis and severity of ischemia with INOCA.” (p. 72 [47]). Additionally, a large number of inferential tests are presented in Table 1, Table 2, Table 3 and Table 4; in recognition of the multiple testing and inflated false discovery rate in those tables, a 0.01 Type I error rate was employed to evaluate *p* values for the between-group frequency results. The presence of diagnosed comorbidities among the participants at the time of evaluation could have influenced the results. Future replication with larger datasets having expanded population representation, and addressing noted limitations, is necessary.

## 5. Conclusions and Recommendations

Our study showed that, among women with signs and symptoms of ischemic heart disease, we observed significant symptom differences between women with and without obstructive CAD. We determined a best-fit model, accurately predicting the presence of no obstructive CAD and, therefore, INOCA based on symptom presentation about 72% of the time. It is important to note that the use of age and symptoms alone for diagnosis of INOCA would not be supported by our findings. Noted limitations, including the heterogeneity of cardiac and non-cardiac symptom presentation, restricts the clinical utility of this unvalidated age- and 9-symptom model. As we have noted above, there is currently no accepted test or risk model for the form of MVD without obstruction (INOCA), leaving a scientific gap in observable factors having discriminatory capacity for this condition. Our pilot findings demonstrate promise for consideration of symptom inclusion in future prediction model development for women with evidence of symptomatic ischemia but no obstruction.

## Figures and Tables

**Table 1:** Baseline characteristics of study population.

	No Obstructive CAD (*n* = 567)	Obstructive CAD (*n* = 349)	*p* Value
Age	55.6 ± 10.8	62.6 ± 11.6	<0.001 ^†^
**Race**			0.180
White	468 (82.5)	275 (78.8)	0.196
American Indian or Alaska Native	1 (0.18)	1 (0.29)	
Asian or Pacific Islander	0 (0.00)	4 (1.15)	
Black or African American	94 (16.6)	66 (18.9)	
Hispanic/Latino	3 (0.53)	2 (0.57)	
Other	1 (0.18)	1 (0.29)	
BMI (kg/m^2^)	30.0 ± 6.8	29.3 ± 6.2	0.135
Total cholesterol	211.9 ± 49.6	214.1 ± 50.7	0.587
HDL-cholesterol	53.0 ± 15.1	48.6 ± 14.4	<0.001 ^†^
LDL-cholesterol	123.9 ± 42.7	129.1 ± 44.6	0.172
**Cardiac risk factors**			
Hypertension	304 (53.7)	237 (68.5)	<0.001 ^†^
Diabetes Mellitus	93 (16.5)	135 (38.8)	<0.001 ^†^
Dyslipidemia	244 (46.5)	222 (68.9)	<0.001 ^†^
Family history of CAD or SCD	364 (65.6)	222 (66.1)	0.942
**Smoking status**			
Never smoked	278 (49.2)	151 (43.3)	0.186
Current or Former smoker	187 (50.8)	198 (56.7)	
Alcohol use within 6 months	79 (14.0)	44 (12.7)	0.689
Current oral contraceptive use	7 (1.3)	2 (0.6)	0.496
Polycystic ovarian disease	41 (7.4)	10 (2.9)	0.005 **
Natural menopause	164 (30.3)	162 (49.5)	<0.001 ^†^
Auto-immune disease	58 (10.4)	42 (12.2)	0.448
History of Depression	155 (27.6)	66 (19.2)	0.004 **
Mitral valve prolapse	98 (17.5)	20 (5.9)	<0.001 ^†^
Migraine	161 (28.8)	57 (16.8)	<0.001 ^†^
**Angina**			
Unstable angina in last 6 weeks	166 (29.6)	144 (42.2)	<0.001 ^†^
Angina at rest	70 (63.6)	36 (45.6)	0.017 *
New onset exertional angina	28 (25.4)	19 (24.0)	0.866
Recent acceleration of angina	16 (14.5)	28 (35.4)	0.001 ^†^
Prior myocardial infarction	54 (9.8)	127 (37.3)	<0.001 ^†^
History of congestive heart failure	35 (6.2)	44 (12.8)	0.001 ^†^

Values are presented as mean ± SD or number (%). * *p* < 0.05; ** *p* ≤ 0.01; ^†^ *p* ≤ 0.001. BMI: body mass index; CAD: coronary artery disease; HDL: high-density lipoprotein; LDL: low-density lipoprotein; SCD: sudden cardiac death.

**Table 2:** Symptom presentation based on absence or presence of obstructive CAD.

Main Symptoms Described at Time of Evaluation	No Obstructive CAD (*n* = 567)		Obstructive CAD (*n* = 349)		*p*-Value
	N	%	N	%	
Pain above the waist	541	95.4	318	91.1	0.011 **
Chest pain	338	59.6	152	43.5	<0.001 ^†^
Chest pressure	321	56.6	149	42.7	<0.001 ^†^
Shortness of breath	303	53.4	144	41.3	<0.001 ^†^
Chest discomfort (general)	394	69.5	176	50.4	<0.001 ^†^
Chest discomfort (heaviness/burning/tenderness)	301	69.3	144	65.4	0.329
Chest tightness	294	51.8	135	38.7	<0.001 ^†^
Fatigue/weakness/faintness	260	45.9	113	32.4	<0.001 ^†^
Palpitations/rapid heart rate	260	45.9	91	26.1	<0.001 ^†^
Arm pain or shoulder pain	251	44.3	139	39.8	0.192 *
Numbness/tingling in arm or hand	212	37.4	104	29.8	0.022 *
Dizziness/lightheadedness	203	35.8	102	29.2	0.043 *
Back pain	185	32.6	88	25.2	0.017 *
Sweating	190	33.5	91	26.1	0.018 *
Neck pain	164	28.9	67	19.2	0.001 ^†^
Headache	141	24.9	67	19.2	0.051
Nausea/vomiting	113	19.9	57	16.3	0.190
Jaw pain	85	15.0	53	15.2	0.925
Abdominal pain	68	12.0	26	7.4	0.033 *
Impending doom	57	10.0	35	10.0	1.00
Cough	99	17.5	52	14.9	0.359

* *p* < 0.05; ** *p* ≤ 0.01; ^†^ *p* ≤ 0.001. CAD: coronary artery disease.

**Table 3:** Location and description of sensation.

Location of Pain	No Obstructive CAD (*n* = 567)		Obstructive CAD (*n* = 349)		*p*-Value
	N	%	N	%	
Central chest	277	48.8	129	37.0	<0.001 ^†^
Center chest/behind breastbone	346	61.0	208	59.6	0.677
Left arm	175	30.9	97	27.8	0.334
Left chest	233	41.1	91	26.1	<0.001 ^†^
Left shoulder	150	26.5	72	20.6	0.048
Left neck	133	23.5	51	14.6	0.001 ^†^
Left hand	94	16.6	53	15.2	0.643
Central neck	91	16.0	32	9.2	0.003 **
Both hands	60	10.6	22	6.3	0.032 *
Right neck	65	11.5	24	6.9	0.029 *
Both shoulders	57	10.0	28	8.0	0.349
Right arm	36	6.3	17	4.9	0.385
Both arms	54	9.5	34	9.7	0.909
Throat pain	82	14.5	33	9.5	0.031
Right shoulder	40	7.0	24	6.9	1.00
Right hand	26	4.6	19	5.4	0.637
Stomach pain	59	10.4	28	8.0	0.248
Right chest	49	8.6	26	7.4	0.620
Lower back	108	19.0	52	14.9	0.127
Middle back	121	21.3	59	16.9	0.104
Upper back	95	16.7	42	12.0	0.056
**Described sensation**					
Pressure	277	65.8	121	58.4	0.078
Discomfort	264	62.7	112	54.1	0.046
Pain	254	60.3	122	58.9	0.795
Tightness	246	58.4	120	58.0	0.931
Aching	127	30.2	63	30.4	1.00
Sharp Knife-Like Pain	113	26.8	42	20.3	0.077
Nausea	101	24.0	46	22.2	0.689
Numbness	100	23.7	57	27.5	0.327
Indigestion	92	21.8	45	21.7	1.00
Burning	64	15.2	45	21.7	0.044 *
Doctor said feeling is heart related	225	54.7	154	74.4	<0.001 ^†^
**Aggravating factors**					
Upper body exertion	144	35.3	75	38.3	0.527
Whole body exertion	202	49.0	97	48.5	0.931
Very hot/cold weather	99	24.4	66	33.2	0.025 *
Sexual activity	51	12.7	24	12.6	1.00
Lower body exertion	240	58.5	114	57.0	0.727
Meals	90	22.2	41	20.7	0.752
Emotions	248	59.8	105	53.0	0.117
**Relieving factors (relieves sensation)**					
Stopping activity	206	49.2	103	50.0	0.865
Rest	276	65.9	133	64.6	0.788
Nitroglycerine	120	28.6	97	47.1	<0.001 ^†^
Antacid meds	61	14.6	30	14.6	1.00
Nothing relieves sensation	81	19.3	35	17.0	0.513
**Change of feeling**					
Feeling wakes you from sleep	195	47.2	90	44.5	0.548
Feeling changes when you take deep breath	111	27.1	53	26.6	0.923
Feeling changes when you press on it	78	19.2	26	13.2	0.084
Feeling changes when you change positions	130	31.5	59	29.9	0.709

* *p* < 0.05; ** *p* ≤ 0.01; ^†^ *p* ≤ 0.001. CAD: coronary artery disease.

**Table 4:** Symptom duration, intensity and response to sensation, frequency.

Characteristic	No Obstructive CAD (*n* = 567)		Obstructive CAD (*n* = 349)		*p*-Value
	N	%	N	%	
**How long does feeling last**					0.029 *
Less than 1 min	34	8.2	11	5.4	
1–5 min	139	33.5	55	27.1	
5–15 min	84	20.2	62	30.5	
15–30 min	65	15.7	23	11.3	
30–60 min	28	6.7	20	9.8	
More than 60 min	65	15.7	32	15.8	
**Usual Intensity of symptoms**					0.109
Tolerable, no relief needed	66	16.1	25	12.5	
Tolerable, relieved with usual measures	178	43.4	83	41.5	
Tolerable, not relieved with usual measures	115	28.0	51	25.5	
Not tolerable, relieved with usual measures	37	9.0	27	13.5	
Not tolerable, not relieved with usual measures	14	3.4	14	7.0	
**Response to sensation**					
Talked to coworker	95	22.1	33	15.3	0.047
Accepted symptoms	238	55.6	104	47.9	0.067
Did nothing to respond to symptoms	121	28.4	39	17.9	0.004 **
Disengaged self from symptoms by doing/thinking something else	178	41.6	72	33.3	0.049
Ignored symptoms	130	30.2	56	25.9	0.271
Redefined symptoms/situation as not threatening	187	43.6	77	35.8	0.062
Sought/seeking medical care due to uncomfortable sensation	398	94.5	199	97.5	0.101
**Average frequency of symptoms**					0.464
Daily	128	31.7	73	37.4	
Weekly	158	39.1	65	33.3	
Monthly	69	17.1	32	16.4	
Yearly	49	12.1	25	12.8	

* *p* < 0.05; ** *p* ≤ 0.01.

**Table 5:** Best Fit Logistic Model of Independent Predictors of INOCA.

Parameter	df	Maximum Likelihood Estimate	Odds Ratio	Lower 95% CI	Upper 95% CI	Wald Chi-sq	*p*
Intercept	1	−0.2556	N/A	N/A	N/A	1.11	0.2916
Age ^1^	1	0.4516	2.468	1.833	3.338	34.95	<0.001 ^†^
Left side chest pain ^2^	1	0.3685	2.090	1.475	2.988	16.78	<0.001 ^†^
Impending doom ^2^	1	−0.2936	0.556	0.335	0.927	5.14	0.023 *
Neck pain ^2^	1	0.2894	1.784	1.172	2.745	7.13	0.008 **
Palpitations ^2^	1	0.4029	2.238	1.557	3.237	18.65	<0.001 ^†^
Chest discomfort ^2^	1	0.3799	2.138	1.465	3.136	15.36	<0.001 ^†^
Left arm pain ^2^	1	−0.3913	0.457	0.305	0.679	14.80	<0.001 ^†^
Bilateral arm pain ^2^	1	−0.3709	0.476	0.278	0.817	7.31	0.007 **
Right hand pain ^2^	1	−0.3902	0.458	0.228	0.929	4.78	0.029 *
Jaw pain ^2^	1	−0.2492	0.607	0.394	0.936	5.10	0.024 *

^1^ Referent group is ≤55. ^2^ Referent group is symptom absent. * *p* < 0.05; ** *p* ≤ 0.01; ^†^ *p* ≤ 0.001.

## Data Availability

The data presented in this study are openly available in NIH/NHLBI’s BioLINCC repository at URL: https://biolincc.nhlbi.nih.gov/studies/wise/, accession reference number HLB00490507a, (accessed on 29 June 2023).
